# What happened after the epidemic? Equine influenza surveillance sheds light on sources and seasonal risk in the United Kingdom

**DOI:** 10.1002/evj.70156

**Published:** 2026-03-05

**Authors:** Fleur Whitlock, John Grewar, Richard Newton

**Affiliations:** ^1^ Equine Infectious Disease Surveillance (EIDS), Department of Veterinary Medicine University of Cambridge Cambridge UK; ^2^ jDATA (Pty) Ltd Sandbaai South Africa

**Keywords:** epidemiology, flu, H3N8, horse, horse importation

## Abstract

**Background:**

The epidemiology of equine influenza (EI) in the United Kingdom has not been systematically described since the 2019 epidemic.

**Objectives:**

To summarise UK EI surveillance (2020–2024), quantify outbreak seasonality and assess movement‐related sources.

**Study Design:**

Retrospective observational analysis of national surveillance and horse importation data.

**Methods:**

Epidemiological data for laboratory‐confirmed EI cases in the United Kingdom were collated. Outbreaks (EI‐infected premises) were defined as one or more laboratory‐confirmed cases on the same premises within a 4‐week period. Monthly outbreak counts were analysed using negative binomial regression with year, calendar‐quarter and ordered quartiles of 1‐month lagged Irish exports to the United Kingdom by equid commodity code. A subset of Q4‐2022 sales‐related EI outbreaks were mapped.

**Results:**

Epidemiological data were available for 149 cases on 126 premises. Outbreaks displayed a repeatable late‐year pattern: Q4 (October to December) accounted for 52% (65/126), with a 3.25‐fold higher per‐month rate than the rest of the year. Over 75% (95/126) of premises reported a new arrival within ≤2 weeks; 56% (28/50) of index new‐arrival cases with recorded origin came from Ireland. Q4 incidence exceeded Q1 (incidence rate ratio [IRR] 6.9, *p* < 0.001) and years 2021–2024 incidence exceeded 2020 (IRRs 4.5–5.6, *p* < 0.001). Adding lagged Irish imports other than pure‐bred breeding animals, improved fit, attenuated the Q4 effect (IRR = 3.9, *p* < 0.001) and identified higher import quartiles as predictors (quartile‐3: IRR 4.5, *p* < 0.001; quartile‐4: IRR 3.7, *p* < 0.001).

**Main Limitations:**

Under‐ascertainment, UK‐wide exposure data versus Great Britain‐only outcomes, COVID‐19 suppression of movements/testing in 2020.

**Conclusions:**

EI in the United Kingdom in 2020–2024 was characterised by a notable October to December risk window and strong links to horse movements. Trade in non‐pure‐bred horses aligns with outbreak timing and partly explains the seasonal excess. Control measures should prioritise vaccination of new arrivals, post‐arrival quarantine and strengthened biosecurity during transport.

## Abstract (de)

**Hintergrund:**

Die Epidemiologie der Pferdegrippe, Equinen Infuluenza (EI), im Vereinigten Königreich (UK) wurde seit der Epidemie von 2019 nicht systematisch beschrieben.

**Ziel der Studie:**

Zusammenfassung der EI‐Surveillance im Vereinigten Königreich (2020–2024), Quantifizierung der saisonalen Ausbrüche und Bewertung der mit Transporten verbundenen Ursachen.

**Studiendesign:**

Retrospektive Beobachtungsanalyse nationaler Überwachungs‐ und Pferdeimportdaten.

**Methoden:**

Es wurden epidemiologische Daten zu laborbestätigten EI‐Fällen im Vereinigten Königreich zusammengestellt. Ausbrüche (mit EI infizierte Betriebe) wurden als ein oder mehrere laborbestätigte Fälle in demselben Betrieb innerhalb eines Zeitraums von vier Wochen definiert. Die monatlichen Ausbruchszahlen wurden unter Verwendung einer negativen binomialen Regression mit Jahr, Kalenderquartal und geordneten Quartilen der um einen Monat verzögerten irischen Exporte in das Vereinigte Königreich nach Equiden‐Zolltarifnummern analysiert. Eine Untergruppe der verkaufsbezogenen EI‐Ausbrüche im vierten Quartal 2022 wurde kartografisch dargestellt.

**Resultate:**

Epidemiologische Daten lagen für 149 Fälle in 126 Einrichtungen vor. Die Ausbrüche zeigten ein wiederkehrendes Muster zum Jahresende: 52% (65/126) entfielen auf das 4. Quartal (Oktober bis Dezember), wobei die monatliche Rate 3,25‐mal höher war als im Rest des Jahres. Über 75% (95/126) der Einrichtungen meldeten innerhalb von ≤2 Wochen einen Neuzugang; 56% (28/50) der Indexfälle von Neuzugängen mit dokumentierter Herkunft stammten aus Irland. Die Inzidenz im vierten Quartal übertraf die des ersten Quartals (Inzidenzratenverhältnis [IRR] 6,9, *p* < 0,001) und die Inzidenz in den Jahren 2021–2024 übertraf die von 2020 (IRRs 4,5–5,6, *p* < 0,001). Durch Hinzufügen von verzögerten Importen aus Irland, die keine reinrassigen Zuchttiere waren, verbesserte sich die Anpassung, der Q4‐Effekt wurde abgeschwächt (IRR = 3,9, *p* < 0,001) und höhere Importquartile wurden als Prädiktoren identifiziert (Quartil 3: IRR 4,5, *p* < 0,001; Quartil 4: IRR 3,7, *p* < 0,001).

**Haupteinschränkungen:**

Untererfassung; britische Expositionsdaten im Vergleich zu Ergebnissen nur für Großbritannien; COVID‐19‐bedingte Einschränkung von Bewegungen/Tests im Jahr 2020.

**Schlussfolgerung:**

Die EI im Vereinigten Königreich in den Jahren 2020–2024 war durch einen auffälligen Risiko‐Zeitraum von Oktober bis Dezember und einen starken Zusammenhang mit Pferdetransporten gekennzeichnet. Der Handel mit nicht reinrassigen Pferden deckt sich mit dem Zeitpunkt des Ausbruchs und erklärt teilweise den saisonalen Anstieg. Kontrollmaßnahmen sollten die Impfung von Neuankömmlingen, die Quarantäne nach der Ankunft und eine verstärkte Biosicherheit während des Transports priorisieren.

## 1 INTRODUCTION

Equine influenza virus (EIV) is a major respiratory pathogen in equids, although in populations where equine influenza (EI) is endemic and vaccination is applied as a preventive measure, clinical signs are typically mild and transient.^1^ However, an epidemic in West Africa in 2019 resulted in over 60,000 fatalities, highlighting the severe impact EI can have on equine populations, as was also demonstrated during the 2007 outbreak in Australia, which led to widespread movement restrictions and eradication costs estimated at around AU $1 billion.^2^, ^3^ Surveillance plays a critical role in monitoring viral evolution, informing vaccine updates and guiding outbreak response. Each year, global surveillance data are reviewed by the Expert Surveillance Panel (ESP) on Equine Influenza Vaccine Composition, a World Organisation for Animal Health (WOAH) initiative.^4^ The scope and quality of EI surveillance vary worldwide due to differences in resources, equine industry structures and disease dynamics. The United Kingdom benefits from a well‐established surveillance programme that facilitates subsidised testing and real‐time epidemiological and virological analysis, aiding outbreak control and minimising industry disruption.

The only current circulating subtype of EIV globally is H3N8 and this was first identified in North America in 1963.^5^ Until the late 1980s, H3N8 evolved as a single lineage before diverging into two antigenically distinct clusters: the American and Eurasian lineages. The American lineage later split into the Kentucky, Argentina and Florida sub‐lineages, with the Florida sub‐lineage further dividing into Florida clade 1 (FC1) and Florida clade 2 (FC2) in the early 2000s and both clades have co‐circulated for over two decades. The last major outbreak of a FC2 virus in the United Kingdom occurred in 2003, while FC1 has since caused significant outbreaks in South Africa (2003), Japan and Australia (2007), India (2008) and South America (2012, 2018).^3^, ^6^, ^7^, ^8^, ^9^, ^10^, ^11^ In 2019, a FC1 virus was identified in the United Kingdom and Europe as a cause of multiple outbreaks for the first time in almost a decade, marking a significant shift in regional EI epidemiology.^12^

EI occurrence is shaped by management and movement patterns as much as by virology. In the United Kingdom, periods of increased EI activity have been described where mixing intensifies seasonally, for example, in unvaccinated non‐Thoroughbreds around summer shows and in vaccinated young Thoroughbreds during the autumn sales.^12^, ^13^ High‐throughput hubs, such as sales yards, dealers, lairage and shared transport, can amplify transmission, particularly in sectors without mandated vaccination or where booster intervals drift beyond recommendations. Outbreaks are also frequently linked to the introduction of infected new arrivals either from within or beyond the United Kingdom, with subsequent onward spread.^12^ Cross‐border movements, including imports, are a recurrent source of introductions globally and may shape the temporal pattern of risk. Against this background, we aimed to use national surveillance data to (i) describe post‐2019 EI activity in the United Kingdom from 2020 through to the end of 2024, (ii) quantify seasonal patterns in EI outbreaks at the premises level and (iii) evaluate movement‐related sources of infection, including live equine import streams by commodity class. Focussing on this post‐epidemic period, which spans COVID‐19‐related reductions in horse movements and the subsequent resumption of sales, trade and gatherings, enables us to assess how EI dynamics have changed and to identify where surveillance, vaccination and biosecurity efforts could be most effectively targeted.

## 2 MATERIALS AND METHODS

### 2.1 Data collection and sources

The Horserace Betting Levy Board (HBLB) EI surveillance scheme enables veterinary practitioners in the United Kingdom to submit upper respiratory tract samples for diagnostic testing for EIV by quantitative reverse transcription PCR (qRT‐PCR) at no cost, utilising a designated laboratory. Typically, samples are nasopharyngeal swabs but may also include nasal swabs, nasopharyngeal washes or tracheal washes. Occasionally EI cases are confirmed positive through high titres on serology (haemagglutination inhibition test) in the absence of vaccination. The HBLB scheme is overseen by epidemiologists from Equine Infectious Disease Surveillance (EIDS) and equine virologists, both affiliated with the Department of Veterinary Medicine at the University of Cambridge. Veterinary diagnostic laboratories and submitting practitioners in the United Kingdom confirming presence of EIV in diagnostic samples tested by agent detection methods are encouraged to contribute positive samples for surveillance and research purposes and provide accompanying epidemiological data. Diagnostic confirmation of EIV infection may have been performed by real‐time qRT‐PCR, insulated isothermal RT‐PCR (iiPCR) or loop‐mediated isothermal amplification (LAMP), depending on the submitting laboratory. qRT‐PCR assays targeted one or more of the matrix (*M*), nucleoprotein (*NP*), haemagglutinin (*HA*), neuraminidase (*NA*) and polymerase (*PB1, PB2, PA*) genes.^14^, ^15^ Some laboratories employed in‐house qRT‐PCR assays for which no validated peer‐reviewed methods are available. iiPCR‐based detection of EIV used an *HA*‐targeted insulated isothermal RT‐PCR assay validated for H3N8 EIV.^16^ LAMP‐based detection followed an *HA*‐targeted RT‐LAMP protocol employed as a point‐of‐care assay for equine respiratory pathogens.^17^

In instances where samples were positive for EIV, with veterinary practitioner and equine keeper permissions, epidemiological data were gathered using EIDS's outbreak form (Figure S1). This study therefore analysed a convenience sample of EI‐positive horses and premises for which diagnostic submissions were made and permission to share epidemiological data was obtained, rather than a random or population‐based sample. Data around the total number of samples tested (by agent detection and serological methods) for EIV were obtained from the laboratory surveillance section of the Equine Quarterly Disease Surveillance report (EQDSR) which is produced by EIDS in collaboration with the Animal and Plant Health Agency (APHA) and the British Equine Veterinary Association (BEVA).

### 2.2 Epidemiological data analyses

Epidemiological data were entered and stored in a PostgreSQL database (PostgreSQL Global Development Group—www.postgresql.org). Exploratory data analysis was conducted using R (R Core Team, 2025) in RStudio version 1.1.463, with additional packages, including RPostgres, dplyr, ggplot2 and tidyr.^18^, ^19^, ^20^, ^21^ Descriptive analyses were performed at both EI‐infected premises and confirmed case levels, with proportions expressed as percentages alongside 95% confidence intervals (CIs) where denominators were available and counts were sufficient to support reliable estimates. Data distribution was assessed visually through histograms and box plots. Depending on the distribution, normally distributed continuous variables were summarised using means and standard deviations, while right‐skewed continuous data were reported using medians, interquartile ranges (IQRs) and ranges. At a national scale, spatial and temporal analyses included the creation of choropleth maps, using QGIS and an outbreak curve, using R studio.^22^

EI‐infected premises (hereafter also referred to as outbreaks) were defined as premises with one or more laboratory‐confirmed cases on the same premises within a 4‐week period. A confirmed case was a horse with a positive agent‐detection test for EIV (qRT‐PCR or LAMP, according to laboratory cut‐offs). In a small number of instances, confirmation was by serology. For temporal analyses, outbreaks were indexed by the date of the first positive sample from that premises. After initial visual assessment, seasonality was described post hoc by comparing EI outbreak rates in Q4 (October to December) with those in all other months using Poisson rate ratios (RRs) with 95% CIs. Rates were defined as outbreaks per unit time per month for 2020–2024 analysis and per quarter for year‐specific estimates. RRs and their 95% CIs were calculated for the full 5‐year data set and for each calendar year separately. To investigate both the seasonal patterns and temporal trends in EI outbreaks, negative binomial regression models were used to account for overdispersion in the outbreak count data. A log‐link function was applied using the glm.nb() function in R (MASS package), with the dependent variable being the monthly count of laboratory‐confirmed EI outbreaks between January 2020 and December 2024, inclusively.^23^ Categorical variables for year and calendar‐quarter (Q1: January to March, Q2: April to June, Q3: July to September, Q4: October to December) were initially included to evaluate baseline temporal patterns.

In addition to assessing seasonality, potential correlations between live equine imports into the United Kingdom and EI outbreaks were explored. Data on live equine imports into the United Kingdom from 2020 to 2024 were obtained from the UN Comtrade database using the comtradr R package, with the identity of specific importing countries of most relevance directed by EI case reported data.^24^ The analyses focussed on using the numbers of live horses reported as exported to the United Kingdom by the exporting countries of interest, recorded using the following relevant commodity codes: 0101 (horses, asses, mules and hinnies; including both breeding and non‐breeding horses), 010121 (horses; pure‐bred breeding animals) and 010129 (horses; other than pure‐bred breeding animals). ‘Pure‐bred breeding animals’ refers to horses of recognised breeds that are classified as pure‐bred for breeding purposes. Code 010129 therefore encompasses all other horses, including most animals moved for competition, leisure and commercial sale that are not recorded as pure‐bred breeding stock at the time of export. For consistency and data completeness, export records reported by the individual exporting countries of interest were used, rather than UK‐reported imports, due to more complete monthly reporting and fewer missing or anomalous values. These export data were assessed to identify anomalies such as extreme outliers or cases where entire annual volumes were aggregated into a single monthly entry.

For each commodity code, monthly import totals were aggregated and joined to the UK EI outbreak data set by year and month. A 1‐month lag was applied to import variables to account for likely delay between horse movement, potential EI exposure and incubation and subsequent laboratory confirmation. Total numbers of imported equids for each commodity code were ranked by numerical volumes and categorised into quartiles (Group 1—lowest values quartile, to Group 4—highest values quartile). Groups were included as ordered categorical predictors in separate models alongside calendar‐quarter and year, with Groups 1–4 representing volume‐based quartiles rather than calendar time. This approach was informed by distributional considerations as monthly import volumes were highly skewed, as confirmed by Shapiro–Wilk tests and varied markedly across commodity codes. Categorisation into quartiles avoided undue influence of extreme values and provided a standardised and comparable scale across codes. A total of five negative binomial regression models were specified. Model 1 included calendar year (2020–2024) and calendar‐quarter (Q1–Q4) only, to describe baseline temporal patterns in EI outbreaks. Model 2 included calendar‐quarter and ordered quartiles of 1‐month‐lagged Irish exports of ‘horses other than pure‐bred breeding animals’ (commodity code 010129), without year, to assess the association between this import stream and outbreak counts (Table 1). Models 3–5 (Table S1) extended this framework by including calendar year, calendar‐quarter and ordered quartiles of 1‐month‐lagged Irish exports for alternative commodity codes: all horses (0101; Model 3), pure‐bred breeding horses (010121; Model 4) and non‐pure‐bred horses (010129; Model 5). As UN Comtrade‐derived data are available only at monthly resolution, shorter lag structures (e.g. 2 weeks) could not be assessed, so lag‐structure sensitivity analysis for Model 2 was undertaken by refitting the 1‐month lag model on an identical observation set and comparing it with an alternative 2‐month lag specification (Table S2). The best‐fitting model was identified based on the lowest Akaike Information Criterion (AIC) value. Incidence rate ratios (IRRs) with 95% CIs and *p*‐values were extracted for interpretation. Model dispersion was assessed using the theta (*θ*) parameter to evaluate overdispersion in the count data. Where potential model instability due to multicollinearity and confounding was suspected (e.g., due to Covid pandemic‐related anomalies in 2020), models excluding year were also tested. Model robustness was evaluated by (i) checking for overdispersion to justify the negative binomial form over a Poisson distribution and using (ii) Cook's distance to identify influential outliers, (iii) zero‐inflated negative binomial (ZINB) comparison to assess the need for a dual‐process model and (iv) jackknife estimation to ensure coefficient stability.

**TABLE 1:** Results of two multivariable negative binomial regression models evaluating factors associated with monthly numbers of equine influenza (EI) outbreaks in the United Kingdom between 2020 and 2024: Model 1 with year and year quarter to assess baseline temporal patterns and Model 2 with year quarter and ordered quartiles for numbers of animals other than pure‐bred breeding horses (commodity code 010129) imported from Ireland the preceding month.

Variable	Model 1 baseline model: Year + quarter (no import data)		Model 2 Irish imports (commodity code 010129) in quartiles with 1‐month lag	
	IRR (95% CI)	*p*‐value	IRR (95% CI)	*p*‐value
Year 2020	1.0	Ref.		
Year 2021	5.58 (1.90–16.40)	0.0018**		
Year 2022	4.48 (1.50–13.34)	0.0072**		
Year 2023	5.40 (1.83–15.90)	0.0022**		
Year 2024	4.93 (1.67–14.60)	0.004**		
Quarter 1 (January to March)	1.0	Ref.	1.0	Ref.
Quarter 2 (April to June)	2.67 (1.07–6.62)	0.034*	1.67 (0.72–3.87)	0.23
Quarter 3 (July to September)	2.57 (1.03–6.39)	0.043*	1.68 (0.74–3.84)	0.22
Quarter 4 (October to December)	6.86 (2.91–16.16)	<0.001***	3.90 (1.79–8.51)	<0.001***
Group 1 import volume quartile			1.00	Ref
Group 2 import volume quartile			1.26 (0.51–3.11)	0.62
Group 3 import volume quartile			4.45 (2.11–9.36)	<0.001***
Group 4 import volume quartile			3.66 (1.72–7.81)	<0.001***
AIC	222		213	

*Note*: Results are shown as IRRs with 95% CIs and *p*‐values. AIC values are included for model comparison and quartiles refer to ranked import volumes, not calendar‐quarters.

Abbreviations: AIC, Akaike Information Criterion; CI, confidence intervals; IRR, incidence rate ratio; Ref., reference category.

*p < 0.05; **p < 0.01; ***p < 0.001. The *p*‐values are two‐sided from negative binomial regression; unstarred values are not significant at *p* < 0.05.

To investigate potential transmission pathways of EI, a subset of laboratory‐confirmed cases for which both a sales origin and receiving premises could be established, were mapped. These movements were compiled from epidemiological data collected during outbreak investigations and included only cases where a clear directional trace could be established from a sale or auction to a confirmed infected premises. Map‐based visualisation was used to illustrate potential epidemiological links between outbreaks and movements were plotted with directional arrows from origin to destination and labels indicated the first sample date at the receiving subsequently EI‐infected premises. County centroids were used to preserve confidentiality. This approach aimed to identify potential clusters of outbreaks sharing a common source or occurring within close spatial–temporal proximity.

## 3 RESULTS

For the 5‐year period 2020 to 2024, laboratory surveillance data presented in the EQDSRs demonstrated that there had been a total of 177 positive diagnoses of EI reported in the United Kingdom. These comprised 1.2% (171/14,468) positive PCR tests, 1.2% (3/248) positive loop‐mediated isothermal amplification (LAMP) tests and 0.3% (3/995) positive serological diagnoses, with epidemiological data available for 149 cases. A summary flow diagram showing how cases and premises contributed to each stage of the analysis is provided (Figure S2).

### 3.1 Descriptive EI‐infected premises and confirmed case data

**FIGURE 1:**
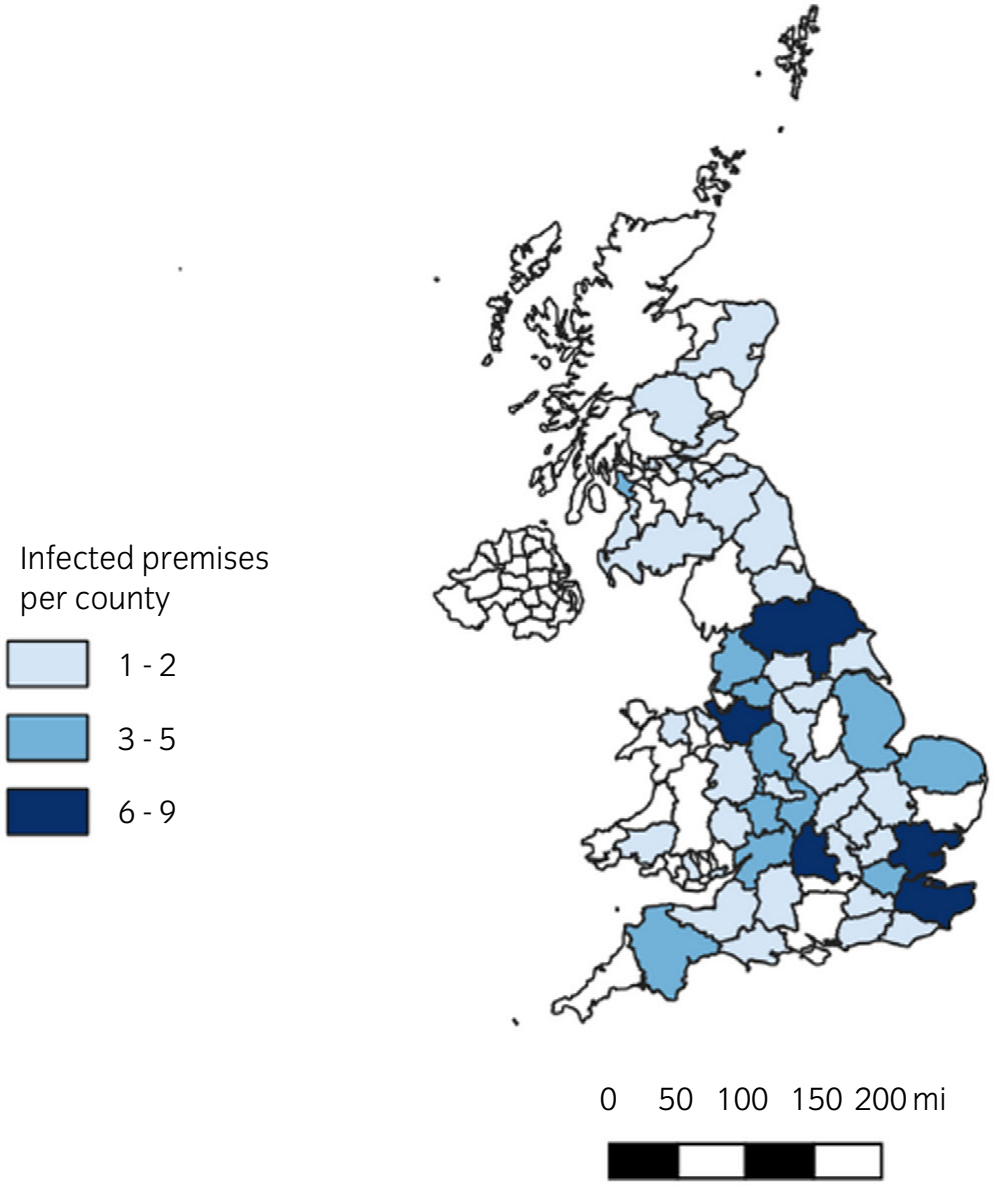
Choropleth map depicting the county‐based locations of equine influenza‐infected premises confirmed between 2020 and 2024 in the United Kingdom.

The 149 confirmed cases with permission given for sharing epidemiological data were located across 126 premises in 53 counties in the United Kingdom (Figure 1). Resident horse population data were available for 99 premises with a confirmed case of EI, among which 58.1% (782/1345; 95% CI 55.4–60.8%) of resident horses were reported by the treating veterinary surgeon as vaccinated against EI. Horse‐level descriptive data relating to breed, sex, vaccination status and associated details are shown in Table 2. Where age was known (*n* = 124), cases had a median age of 4 years (IQR 3–8 years, range 3 months to 24 years). The specific breed types with the highest number of confirmed EI cases were Cobs 16.1% (24/149; 95% CI 10.8–23.2%) and Irish Sports Horses (13.4%, 20/149; 95% CI 8.6–20.2%) (see Table S3 for details of breed type of confirmed cases). Vaccinated confirmed EI cases were identified on 8.7% (11/26; 95% CI 4.7–15.4%) of EI‐infected premises and 8.1% (12/149; 95% CI 4.4–14.0%) of confirmed cases were vaccinated against EIV. Where data were available, sampling methods included nasopharyngeal or nasal swabs (144/151), nasopharyngeal wash (4/151), tracheal wash (1/151) and two cases were confirmed positive through haemagglutination inhibition serology, in absence of vaccination (2/151).

**TABLE 2:** Proportional and frequency data for horse factors for UK equine influenza cases occurring between 2020 and 2024.

Factor	Category	Frequency	Percentage (95% CI)
General breed (*n* = 149)	UK‐native pony breeds	39	26.2 (19.5–34.1)
	Sports horses	36	24.2 (17.7–32.0)
	UK‐native horse breeds	34	22.8 (16.5–30.6)
	Undefined	28	18.8 (13.0–26.2)
	Crossbreeds	5	3.4 (1.2–8.1)
	Non‐UK native horses	6	4.0 (1.7–8.9)
	Donkey	1	0.67 (0.04–4.2)
Sex (*n* = 149)	Mare	63	42.3 (34.3–50.6)
	Gelding	59	39.6 (31.8–48.0)
	Undefined	27	18.1 (12.5–25.5)
Influenza vaccination status (*n* = 149)	Unvaccinated	110	73.8 (65.9–80.5)
	Undefined	23	15.4 (10.2–22.5)
	Vaccinated	12	8.1 (4.4–14.0)
	Lapsed vaccinated	4	2.7 (0.9–7.2)
Class of last influenza vaccination (*n* = 11)	V2 (and >14 days beyond)	3	27.3 (7.3–60.7)
	V3	1	9.1 (4.8–42.9)
	Booster (6 months)	1	9.1 (4.8–42.9)
	Booster (12 months)	6	54.5 (2.5–81.9)

	Class of last influenza vaccination	Total	Median (IQR)
Days since last influenza vaccination^a^	V2 (and >14 days beyond)	2	130.5 (127.8–133.3)
	V3	2	78 (43.5–112.5)
	Booster (6 months)	1	32
	Booster (12 months)	5	317 (82–361)
	Vaccine classes combined	10	130.5 (53.5–274.5)

*Note*: V2, second dose of the primary course; V3, first booster after the primary course.

^a^ Number of days between the date of last vaccination and the date of being a confirmed case of influenza.

**FIGURE 2:**
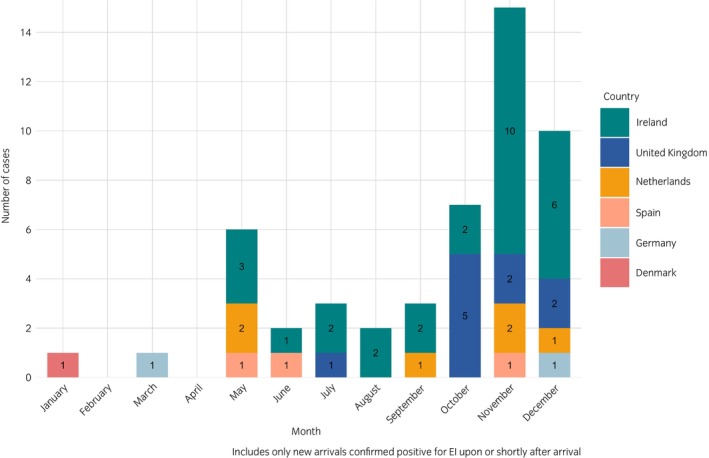
Stacked bar chart showing the reported country of origin (where known) of newly arrived horses that were confirmed equine influenza (EI) cases in the United Kingdom, by month. Bars display cumulative counts across 2020–2024 (*n* = 50).

Table 3 presents descriptive data on EI‐infected premises, categorised by premises type, biosecurity measures adopted and recent reported horse movements on and off the property. Of the 126 infected premises, 75.4% (95/126; 95% CI 66.8–82.4%) reported receiving a new arrival within 2 weeks of a confirmed EI case and among these, 73.7% (70/95; 95% CI 63.5–81.9%) had new arrivals that were laboratory‐confirmed cases. When reported, 75% (45/60; 95% CI 61.9–84.9%) of infected premises did not quarantine new arrivals. The origin of new arrivals was disclosed for 66.3% (63/95; 95% CI 55.8–75.5%) of the infected premises that reported receiving one, and of these, 79.4% (50/63; 95% CI 67.0–88.1%) of the new arrivals were also confirmed cases (Figure 2). Considering seasonality, 56.8% (54/95; 95% CI 46.3–66.8%) of recent (≤2 weeks) new arrivals occurred in Q4 months compared with 43.2% (41/95; 95% CI 33.2–53.7%) in Q1–Q3 months combined; the arrival was a confirmed case in 60% (42/70; 95% CI 47.6–71.3%) in Q4 versus 40% (28/70; 95% CI 28.7–52.4%) in Q1–Q3 combined. The known countries of origin of new arrivals with confirmed EI included: 56% from Ireland (28/50), 20% from within the United Kingdom (10/50), 12% from the Netherlands (6/50), 6% from Spain (3/50), 4% from Germany (2/50) and 2% from Denmark (1/50).

**TABLE 3:** Descriptive data for UK premises confirmed with equine influenza positive horses between 2020 and 2024 (data available for *n* = 126 infected premises).

Factor	Category		Total	Percentage (95% CI)
Premises type (*n* = 126)	Private		46	36.5 (28.3–45.6)
	Undefined		28	22.2 (15.5–30.7)
	Livery		27	21.4 (14.8–29.8)
	Competition yard		9	7.1 (3.5–13.5)
	Riding school		7	5.6 (2.5–11.5)
	Stud		5	4.0 (1.5–9.5)
	Sale/sales yard		3	2.4 (0.6–7.3)
	Training		1	0.8 (0.04–5.0)
Biosecurity measures on infected premises	Isolation facilities available (*n* = 63)	Yes	20	31.7 (20.9–44.8)
		No	43	68.3 (55.2–79.1)
	New arrivals subject to a quarantine period (*n* = 60)	Yes	15	25.0 (15.1–38.1)
		No	45	75.0 (61.9–84.9)
	Horses on premises share equipment (*n* = 46)	Yes	31	67.4 (51.9–80.0)
		No	15	32.6 (20.0–48.1)
	Horses on premises share tack (*n* = 42)	Yes	13	31.0 (18.1–47.2)
		No	29	69.0 (52.8–81.9)
New arrivals (non‐resident) onto infected premises (*n* = 126)	Within 2 weeks^a^ and across all quarters		95	75.4 (66.8–82.4)
	Within 2 weeks^a^ and between Q1 and Q3		41	32.2 (24.5–41.5)
	Within 2 weeks^a^ and during Q4		54	42.9 (34.1–52.0)
New arrivals reported that were also confirmed to be a positive case (*n* = 126)	Within 2 weeks^a^ and across all quarters		70	55.5 (46.5–64.3)
	Within 2 weeks^a^ and between Q1 and Q3		28	22.2 (15.3–30.5)
	Within 2 weeks^a^ and during Q4		42	33.3 (25.2–42.3)

^a^ Weeks preceding a confirmed case on a premises.

**FIGURE 3:**
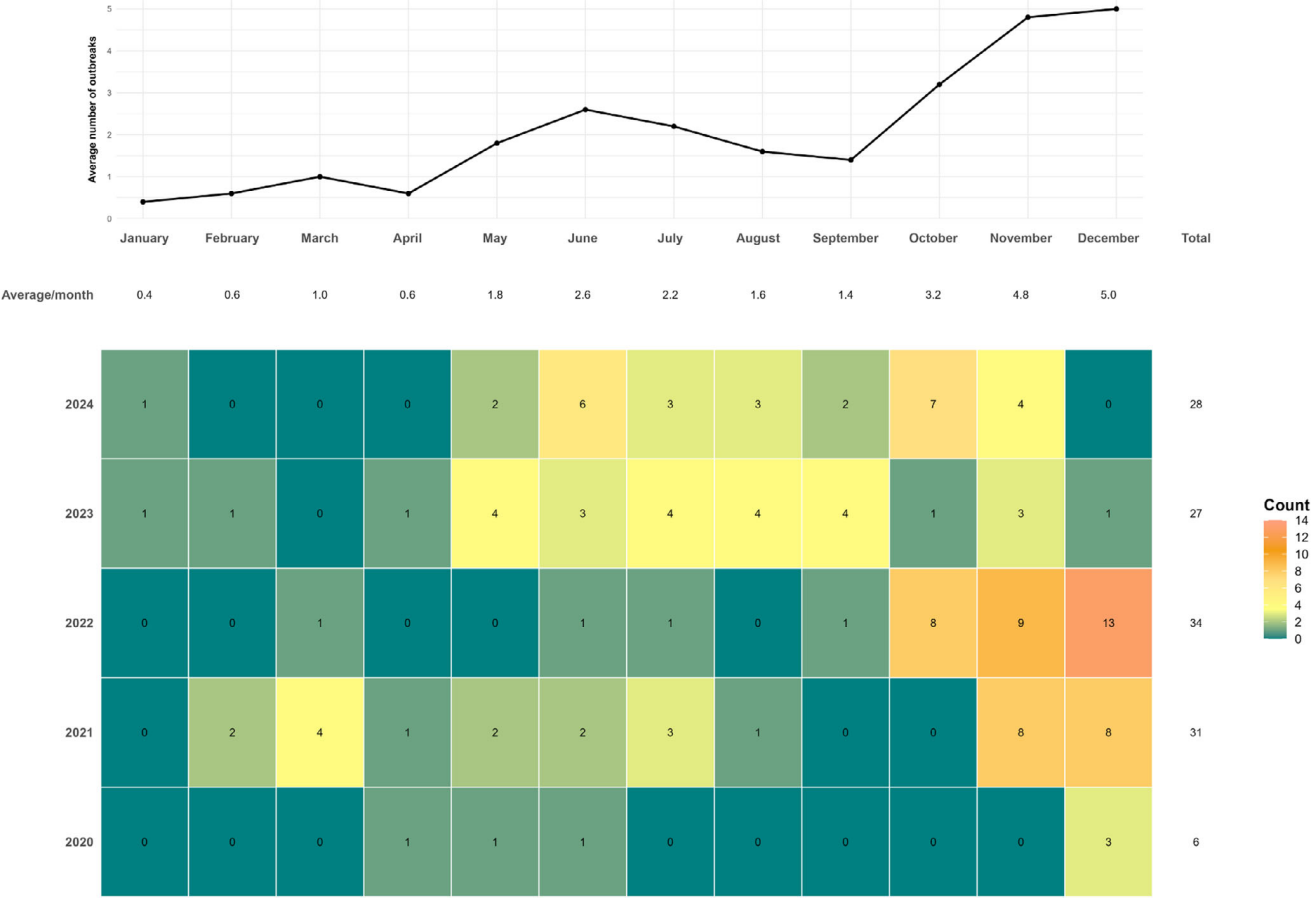
Equine influenza reported outbreaks in the United Kingdom, 2020–2024. Heatmap shows monthly counts by year using the first diagnostic sample date; right column = annual totals; bottom row = mean per calendar month across years; bottom‐right = grand total. Top panel plots the monthly mean (seasonality). Colours increase green → yellow → orange → red with count.

**FIGURE 4:**
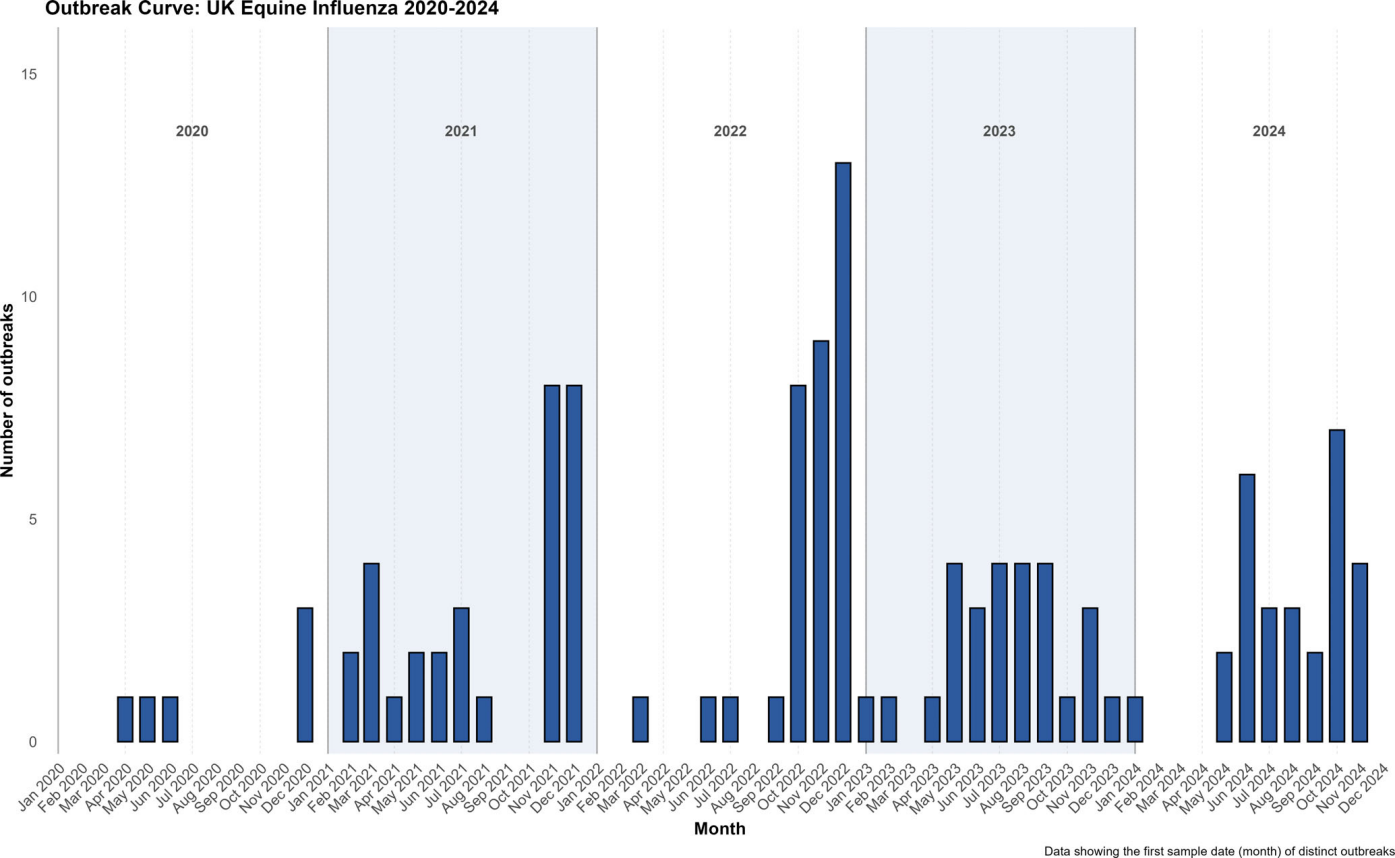
An outbreak curve representing the numbers of monthly EI outbreaks based on date of diagnostic sampling confirmed in the United Kingdom for the 5‐year period between January 2020 and December 2024 (*n* = 125).

A summary of monthly and annual EI outbreak numbers based on date of diagnostic sampling and the average number of outbreaks per month of the year in the United Kingdom are presented in Figure 3 and as an outbreak curve in Figure 4, for the 5‐year period between January 2020 and December 2024. Overall, Q4 accounted for 52% of outbreaks (65/126; 95% CI 42.9–61.0), with a 3.2‐fold higher per‐month rate than the rest of the year (4.3 outbreaks/month vs. 1.4 outbreaks/month; RR 3.2, 95% CI 2.22–4.61). In 2020, outbreaks were relatively rare, but from 2021 onwards there was a clear rise, especially in the latter months of the year, with November and December showing significant increases in 2021 (16 outbreaks) and 2022 (22 outbreaks) (Figures 3 and 4). By year, the Q4 share and corresponding per‐quarter RR were: 2020 = 50% (3/6; RR 3.0, 95% CI 0.4–22.40); 2021 = 51.6% (16/31; RR 3.2, 95% CI 1.5–7.0); 2022 = 88.2% (30/34; RR 22.5, 95% CI 7.9–87.9); 2023 = 19.2% (5/27; RR 0.7, 95% CI 0.2–1.9) and 2024 = 39.3% (11/28; RR 1.9, 95% CI 0.8–4.34). Five‐year monthly averages showed the same seasonal gradient, with Q1 low (≤1), Q2 and Q3 intermediate (0.6–2.6) and Q4 consistently higher (>3 outbreaks/month).

Multivariable negative binomial regression modelling was used to evaluate the simultaneous effects of year and calendar‐quarter on the number of reported EI‐infected premises confirmed in the United Kingdom from 2020 to 2024 (Model 1 in Table 1). Compared to 2020, the incidence rate of EI‐infected premises was significantly higher in every subsequent year (IRR 4.48–5.58; *p* = 0.0018–0.0072), suggesting a consistent level of increased EIV activity in subsequent years relative to 2020. In terms of seasonal patterns, the incidence rate of confirmed EI‐infected premises relative to that seen in Q1, was moderately higher in both Q2 and Q3 (IRR 2.57–2.67; *p* = 0.034–0.043), but markedly higher in Q4 (IRR 6.86; *p* < 0.001), consistent with the pattern of averaged monthly EI summarised in Figure 3. These findings highlight a seasonally varying pattern in EI outbreak risk in the United Kingdom, which is lowest in the first few months of the year, increases during spring and summer and peaks in the last 3 months of the year.

### 3.2 Investigating potential sources of EIV

**FIGURE 5:**
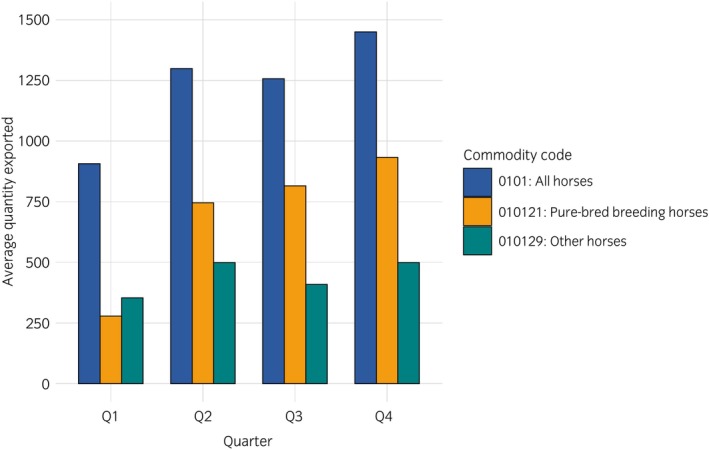
Average quarterly horse movements from Ireland to the United Kingdom (2020–2024), grouped by commodity code. Values represent the average number of horses exported per quarter over the 5‐year period, based on Irish‐reported exports to the United Kingdom from the UN Comtrade database.

Between 2020 and 2024, Ireland, as well as being the most commonly reported origin for new arrivals that subsequently tested positive for EI within 2 weeks of arrival (Figure 2), was also the largest exporter of horses to the United Kingdom, with a total of 25,881 horses recorded for the period and a median of 6095 (IQR 5349–6276) horses per year. The volume of horses from Ireland far exceeded those from other trading partners and showed a consistent temporal pattern across years, and clear seasonal variation was also evident within individual years, with the highest average export volumes occurring in Q4 months (Figure 5). Therefore, further analyses focussed on horse exports recorded from Ireland as it was the leading horse exporting country to the United Kingdom, as well as the most frequently identified source of newly arrived EI‐positive horses in UK outbreak investigations.^24^, ^25^

A best‐fitting multivariable negative binomial regression model (Model 2 in Table 1) included calendar‐quarters and ordered quartiles for animal numbers other than pure‐bred breeding horses (commodity code 010129) imported from Ireland for the preceding month, but excluded year. In this model, outbreak counts were significantly increased in months in which import volumes the preceding month were in quartile Groups 3 and 4 compared to quartile Group 1 (IRR 3.66–4.45; *p* < 0.001). Calendar‐quarter remained a significant predictor, with Q4 remaining associated with higher outbreak counts relative to Q1, although the IRR of 3.9 (*p* < 0.001) was notably reduced compared to the value of 6.86 (*p* < 0.001) in model 1 that included year. In the same model, the incidence risks of EI in Q2 and Q3 were not statistically significantly different from Q1 (IRR 1.67–1.68; *p* = 0.22–0.23). Results from other models incorporating different Irish import metrics (current month commodity terms 0101, 010121, and 010129), along with year and quarter, are shown in Table S1. In lag‐structure sensitivity analyses for Model 2 (fitted on an identical observation set), the 1‐month lag specification showed better model fit (AIC 213) and stronger associations for higher import quartiles (Groups 3 and 4) than the 2‐month lag model (AIC 226), in which effects were attenuated and only the highest import quartile remained significant (Table S2).

Additional diagnostics supported model adequacy and robustness. The negative binomial model was favoured due to significant overdispersion (*p* = 0.02). Cook's distance identified one influential outlier in the Quarter 1 baseline (a higher‐than‐predicted EI outbreak count); its removal did not alter the direction or significance of the primary predictors. ZINB comparisons were non‐significant, confirming the standard model's fit and jackknife resampling confirmed high estimation stability, with Quarter 4 and lagged trade data quartiles 3 and 4 remaining significant across all iterations.

### 3.3 Epidemiologically linked outbreaks

**FIGURE 6:**
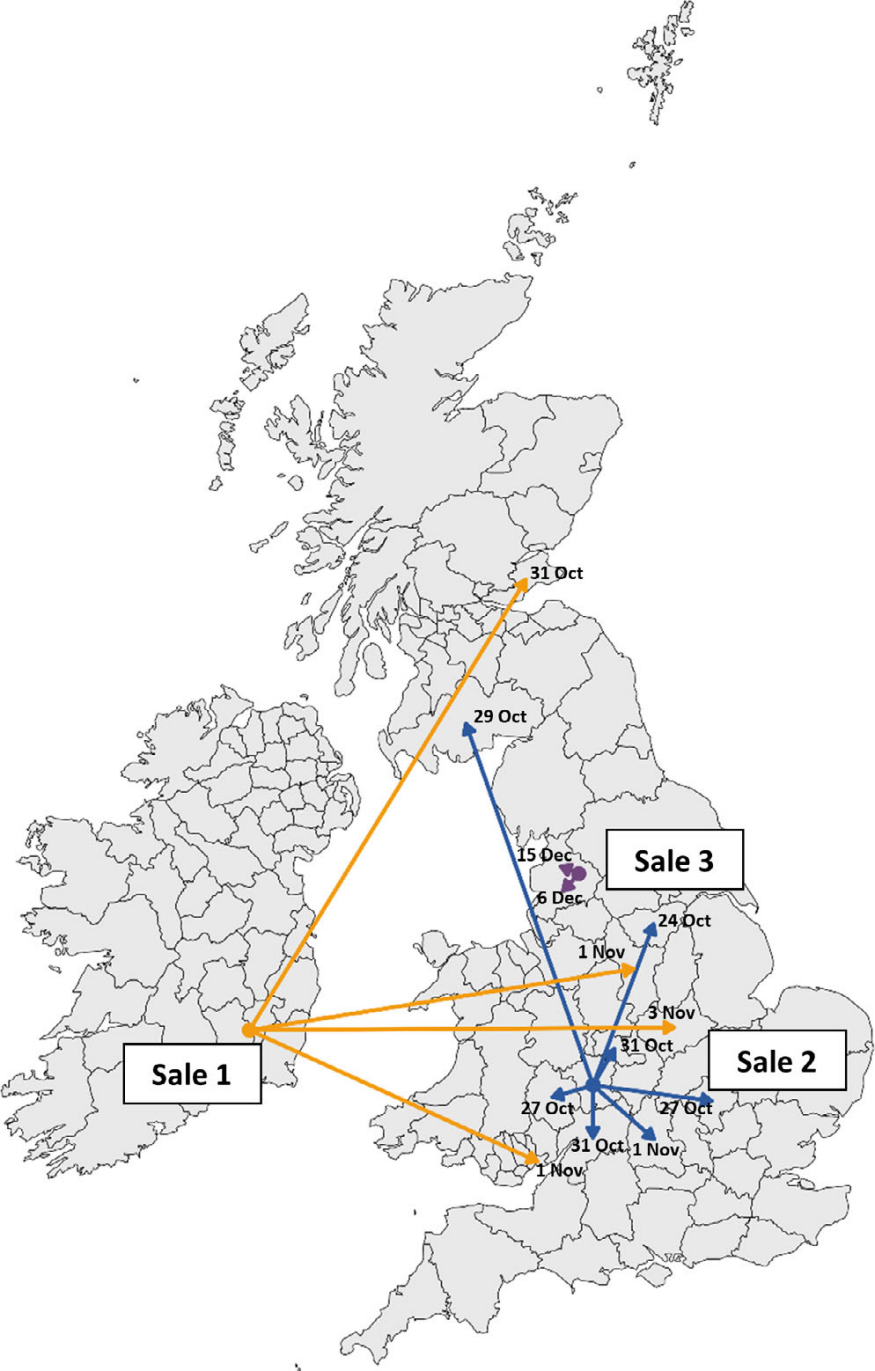
Map of the United Kingdom and Ireland, showing sales origins and premises destinations of equine influenza (EI) confirmed positive cases during quarter four in 2022. Dates represent the sampling dates of the first EI‐positive samples obtained from the receiving premises, locations of which are plotted as county centroids and do not indicate the exact locations of infected premises. Only cases for which both sales origin and destination data were available are presented and this does not represent all EI cases detected during this period.

In Q4 of 2022, a subset of 13 EI‐positive premises was identified as having movement data linking them to several known sales origins, although there was no clear evidence of direct links between the sales themselves. These connections are shown in Figure 6, with arrows representing horse movements from three separate sales venues, one located in Ireland and two in England in Worcestershire and Lancashire, to receiving premises that then confirmed newly arrived horses as EI cases. The sample date at the receiving premises is displayed to indicate the temporal distribution of cases. All mapped movements occurred within a 6‐week period between late October and early December 2022. In several instances, multiple confirmed cases were associated with a single sales origin, suggesting common‐source exposure events. Notably, movements from the Irish sale were linked to EI cases in geographically diverse UK regions, including Scotland, Wales, and the English Midlands, illustrating the potential for long‐distance dissemination of EIV via horse movements.

## 4 DISCUSSION

Following the 2019 European EI epidemic, the first FC1 incursion into the region for almost a decade, EI activity detected in the United Kingdom fell to very low levels in 2020 and subsequently increased in 2021 and was maintained through to the end of 2024. Interpretation of true EI activity in 2020 requires caution as national COVID‐19 restrictions greatly limited equine gatherings, travel and sales, and for a short period that year constrained veterinary activity, sampling and laboratory testing. These restrictions likely reduced true exposure (fewer mixing opportunities) but also lowered ascertainment (fewer examinations/tests conducted for respiratory disease). However, in most years between 2020 and 2024 a repeatable late‐year pattern emerged, with Q4 accounting for around 52% of EI outbreaks, suggesting a seasonal risk window. Previous studies of the 2019 UK epidemic using geographical, epidemiological and whole‐genome data reported a single FC1 introduction from the USA into Europe, followed by multiple independent introductions from Europe into the United Kingdom, rather than sustained local persistence.^26^ Onward spread in 2019 largely occurred in the spring and summer, consistent with transmission amplified at equine gatherings in a population with low vaccine coverage and little prior natural exposure to FC1 EIV.^12^ The reasons as to why the earlier and more prolonged pattern of EI seen in 2019 differed from subsequent years are not clear. However, this may relate to the opportunity that presented in late 2018 to early 2019 with the incursion of a FC1 strain of H3N8 virus that a relatively larger proportion of the UK horse population was susceptible to at that time than has been the case in subsequent years. The more predominant autumn patterns seen in 2020–2024 likely reflect the mixing and movement of a particularly susceptible part of the equine population, thereby increasing the opportunity for onward transmission, manifestation of clinical disease and diagnostic confirmation through passive surveillance.

The composition of infected premises from 2020 to 2024 aligns with movement‐mediated risk late in the year. Of all outbreaks, a greater proportion were Q4 events with a recent non‐resident arrival compared to equivalent events in Q1–Q3 combined (43% vs. 33%; 54/126 vs. 41/126), and the new arrival being the confirmed case was likewise more common in Q4 than Q1–Q3 combined (33% vs. 22%; 42/126 vs. 28/126). These findings are consistent with descriptions from 2019 that horse movements and co‐mingling were central to EI incursions.

Negative binomial models corroborated the descriptive seasonality. In the baseline model with year and calendarquarter (Model 1), incidence in Q4 was markedly higher than in Q1 after adjusting for year, and years 2021–2024 each showed higher incidence than 2020. Including 1‐month‐lagged Irish exports of non‐pure‐bred horses as ordered quartiles (Model 2) improved model fit, reduced the size of the Q4 seasonal effect but did not eliminate it and identified higher import volumes as independent predictors of monthly outbreak counts. After accounting for imports, Q2 and Q3 were not significantly different from Q1 and notably, only non‐pure‐bred horses showed a consistent association; whereas total imports and those of pure‐bred breeding horses only did not. This suggests that the non‐pure‐bred segment of equine trade aligns most closely with UK EI outbreak occurrence. Lag sensitivity analyses supported the prespecified 1‐month lag as the most informative time window in these data. When a 2‐month lag was applied, associations between higher import‐volume quartiles and outbreak counts were attenuated and overall model fit worsened, consistent with importation‐to‐detection processes typically operating within weeks rather than months (i.e., onward contact and amplification shortly after arrival, followed by veterinary assessment and laboratory confirmation, as would be expected with EI's short incubation period). These findings strengthen confidence that the observed association is not an artefact of a single modelling choice, while acknowledging that monthly trade data resolution limits evaluation of shorter lags.

Sensitivity analyses confirmed that the associations between 1‐month lagged Irish trade quartiles and EI outbreak counts were robust and the final model successfully accounted for overdispersion and zero‐counts without requiring zero‐inflation. Despite one influential observation in the baseline first quarter category, the stability of the IRRs during jackknife testing, particularly for the most significant predictors, suggested that the observed associations between Irish trade lags and EI outbreak counts are statistically stable features of the data.

Consistent with this, among UK new arrivals that were laboratory‐confirmed EI cases and had country of origin recorded, over half originated from Ireland, with the majority occurring in Q4 and where recorded, these were invariably unvaccinated non‐Thoroughbreds. This profile is consistent with the association identified by modelling, with the trade in non‐pure‐bred horses and with the late‐year increase.

Together, these findings indicate that the evident EI seasonality persisted after adjusting for trade intensity but that trade in non‐pure‐bred horses could partially explain the EI outbreak numbers seen in Q4. A subset of EI cases confirmed in the United Kingdom in late 2022 illustrated how high‐throughput hubs in the form of horse sales may seed geographically dispersed outbreaks. Traceable links from one Irish and two English sales venues to multiple receiving premises over a short interval were consistent with a hub‐and‐spoke dissemination, with lairage and shared transport as plausible conduits. The clustering of outbreaks in time and with the origin of multiple recently moved cases being linked to the same sales venues, strongly suggests common‐source exposures. Although only cases with both origin and destination data could be mapped and further unrecorded movements and unsampled cases cannot be excluded, the findings suggest that enhancing biosecurity targeted at sales, during transport and immediately post‐arrival is warranted. Movements associated with the Irish sale were linked to cases across multiple UK regions, highlighting the potential for high‐volume trade routes to facilitate long‐distance dissemination of EIV within the United Kingdom. Previous UK and international experience of imports and high movement hubs precipitating or amplifying EI outbreaks have been noted. Rapid EI spread in Newmarket in 2003 impacted more than 1300 racehorses across 21 training yards, highlighting the role of dense, integrated contact networks in EI transmission.^6^ Import‐linked introductions precipitated national outbreaks in; South Africa in 2003–2004 and were traced to imported, subclinically infected horses; Australia in 2007 due to a quarantine breach resulting in extensive spread amongst a naïve population; and in Japan in 2007 within racing facilities. In South America in 2012 transmission of FC1 EI infection across multiple countries was also associated with horse movements and gatherings.^8^, ^27^, ^28^ These events reinforce the importance of traceability in outbreak investigation and surveillance, as data gaps underscore challenges in establishing causality and quantifying risk. Future integration of whole‐genome sequencing and phylodynamic tools (e.g., TransPhylo, which has recently been applied to transmission of *Streptococcus equi* in horses) linked to detailed movement records, would help differentiate coincidental from true epidemiological connections and sharpen targeting of biosecurity measures at high‐risk transmission hubs such as sales and auctions.^29^

Features of these outbreaks are more compatible with repeated introductions and shorter transmission chains than with sustained endemic circulation, and several features of the more recent 2020–2024 UK surveillance data also support a repeated introduction‐focused interpretation. Firstly, activity was seasonally concentrated, with a clear Q4 peak and minimal Q1 activity. Secondly, outbreaks were frequently movement‐linked: approximately three‐quarters of infected premises reporting a non‐resident new arrival within ≤2 weeks and in many of those the arriving horse was the index case. Thirdly, horse trade intensity provided an external correlate of risk: Irish‐reported exports of non‐pure‐bred horses, lagged by 1 month, were associated with higher UK monthly outbreak counts, which reduced, but did not eliminate, the Q4 seasonal effect, consistent with movement intensity partially explaining the seasonal rise. The partial confounding of the Q4 seasonal effect by Irish imports suggests that there are still significant residual effects explained by the identified seasonality, perhaps linked to local transmission related factors following the international incursions. These are obviously not otherwise captured within the limited data available for regression modelling. If EI were truly endemic (always present at a low, expected level) in the United Kingdom, more consistent year‐round transmission would be expected, alongside weaker association with imported new arrivals.

### 4.1 Surveillance performance and study limitations

Surveillance of EI in the UK benefits from subsidised testing, national coverage and integrated epidemiology and virology workflows, which together enhance case detection and rapid characterisation. However, several limitations require consideration. Firstly, under‐ascertainment of cases and variable practitioner engagement are likely, particularly when case presentations are mild. Under ascertainment of cases inevitably reduces the amount of EI detected through surveillance, although it is considered likely that the overall pattern of mostly low level and infrequent disease detection with occasional clusters of increased EI detection seen in the period, would not be fundamentally altered. Missed detections of mild or subclinical disease, due to their inherent reduction in transmissibility through lower EI virus shedding, would be less likely to misrepresent the true pattern of disease. Secondly, spatial outputs used county‐centroid masking to prioritise confidentiality but may smooth fine‐scale clustering. Thirdly, because the data set reflects a convenience sample of voluntarily submitted cases, it is unlikely to be fully representative of all EI infections or premises types in the United Kingdom. Alongside this, importation summaries were assembled from exporter‐reported trade data for completeness and consistency and this introduced a scope mismatch because export data were UK‐wide, that is, Great Britain and Northern Ireland (NI) whereas no NI EI outbreaks were reported through the UK surveillance system, although the system has been designed to encourage EI surveillance in NI. Such mismatch could dilute associations because missed EI cases in NI reduce the evidence linking EI with horse movements between Ireland and Great Britain via NI. However, given the history of an Irish origin of many confirmed EI cases among new arrivals in this data set, this limitation would be more likely to attenuate evidence for such links than to generate a spurious association in the opposite direction. Interpretation is nonetheless constrained by limited routinely available data on EI occurrence in Ireland compared with some other European countries (e.g., the United Kingdom and France).^30^

Fourth, import volumes and EI outbreaks both peak in Q4, so the observed association between higher import quartiles and increased outbreak counts may be partially confounded by season; the models cannot fully separate the effect of imports from other Q4‐specific factors (e.g., increased within‐UK movements or mixing or variations in disease prevention measures), and the import IRRs should therefore be interpreted as reflecting correlated seasonal patterns rather than a definitive primary causal effect of trade alone. Finally, genomic transmission data were not analysed here, so it is not possible to formally distinguish repeated introductions with short transmission chains from short‐range between‐premises spread and the movement‐linked signals should be interpreted as consistent with introduction‐driven dynamics rather than definitive proof. The COVID‐19 context further complicates inference: Irish‐to‐UK horse movements were lowest in 2020, and EI activity was minimal. Using 2020 as the regression reference year therefore captured pandemic‐related suppression of both exposure and detection in addition to underlying infection pressure.

### 4.2 Priorities for prevention and control

Given the identified late‐year risk window, the frequency of movement‐linked introductions and documented gaps in vaccination and quarantine (74% [110/149] of confirmed cases unvaccinated; 75% [45/60] of reporting premises not quarantining new arrivals), prevention and control should prioritise vaccination of all resident horses and new arrivals, and movement controls that interrupt introduction and early within‐premises spread. These findings mirror those of previous studies, yet similar gaps persist because vaccination and quarantine measures remain largely voluntary and uptake has been limited despite repeated advisories.^31^, ^32^, ^33^, ^34^ At points of sale or transfer, vaccination status (product and dates) should be verified and unvaccinated or overdue horses refused entry to a premises or managed at separate facilities (remembering that EIV has been demonstrated to spread over extended distances of hundreds of metres in certain optimal weather conditions).^35^ Vaccinated newly purchased or returning horses should be quarantined for at least 14 days (ideally 21 days) with daily temperature monitoring and rapid veterinary involvement and diagnostic testing applied to any pyrexic or coughing horse. On premises, sharing of tack and equipment should be avoided and handler movement managed to prevent cross‐contact between quarantined and resident groups.

During transport and lairage, horses should be confirmed to be well and non‐pyrexic immediately before transportation and having had no contact with horses displaying clinical signs that may be infectious in origin. Vehicles and boxes should be cleaned and disinfected between loads, consignments not mixed, time in shared airspaces minimised and ventilation optimised; comprehensive movement logs (origin, stops, lairage, vehicle/box identifiers) should be maintained to support rapid trace‐back. Sales operators should provide clear post‐sale health advice and a new arrival biosecurity checklist, with buyer follow‐up at 7–10 days to capture post‐health status. These measures directly target pathways repeatedly implicated in EI spread, that is, recent introductions, high‐throughput sales/transport environments and suboptimal vaccination, thereby reducing the seasonal surge and limiting onward amplification on and beyond infected premises.

## 5 CONCLUSION

EI activity in the United Kingdom since the prolonged 2019 epidemic is best explained by recurrent FC1 EIV introductions that repeatedly cluster in the fourth quarter of the year, with transmission commonly amplified where vaccination and quarantine are suboptimal. Descriptive patterns and negative binomial modelling show that non‐purebred trade‐linked movements exert an independent and substantive effect on the seasonal rise in EI seen in the United Kingdom. Measures should therefore prioritise pre‐movement controls such as vaccination and biosecurity at sales, during transport and immediately post‐arrival and not forgetting the receiving premises' resident populations will also benefit from applying additional controls and vaccination to them.

## FUNDING INFORMATION

FW and RN are supported through a combined contribution to Equine Infectious Disease Surveillance (EIDS) from the Horserace Betting Levy Board (HBLB), the Racehorse Owners' Association (ROA) and the Thoroughbred Breeders' Association (TBA). Some of the equine influenza samples may have been run under the free HBLB testing scheme.

## CONFLICT OF INTEREST STATEMENT

The authors declare no conflicts of interest.

## AUTHOR CONTRIBUTIONS

**Fleur Whitlock:** Conceptualization; investigation; writing – original draft; methodology; validation; visualization; writing – review and editing; software; formal analysis; project administration; data curation. **John Grewar:** Writing – original draft; methodology; validation; visualization; writing – review and editing; software; formal analysis; data curation; supervision. **Richard Newton:** Conceptualization; investigation; funding acquisition; writing – original draft; methodology; validation; visualization; writing – review and editing; formal analysis; data curation; supervision; resources.

## DATA INTEGRITY STATEMENT

Fleur Whitlock had full access to all the data in the study and takes responsibility for the integrity of the data and the accuracy of data analysis.

## ETHICAL ANIMAL RESEARCH

This work was approved by the University of Cambridge's Department of Veterinary Medicine Ethics and Welfare Committee (CR867).

## INFORMED CONSENT

Not applicable.

## Supporting information


**Figure S1:** Example of an outbreak form used to gather epidemiological data on equine influenza outbreaks in the United Kingdom from 2020 to 2024.

**Figure S2:** Flow diagram showing how 177 laboratory‐confirmed EI‐positive samples (2020–2024) relate to 149 confirmed cases with epidemiological data, 126 EI‐infected premises and the subsets used for premises‐level and movement‐linked analyses.

**Table S1:** Models 3–5 examine the effect of import volume from Ireland across three commodity codes—0101 (all horses), 010121 (pure‐bred breeding horses) and 010129 (non‐purebred horses)—converted into quartile‐based groups (Group 1 = lowest, Group 4 = highest volume).

**Table S2:** Sensitivity analysis of lag structure for Irish imports (commodity code 010129; non‐pure‐bred horses) and monthly numbers of equine influenza (EI) outbreaks in the United Kingdom, 2020–2024 (restricted to *n* = 58 months with complete lagged import data).

**Table S3:** Frequency data for breed type of UK equine influenza cases occurring between 2020 to 2024.

## Data Availability

The data that support the findings of this study will be openly available at https://github.com/JDATA-EIDS/ei_uk_postepidemic.
