# Supplementary material for: What happened after the epidemic? Equine influenza surveillance sheds light on sources and seasonal risk in the United Kingdom
Source: Equine Vet J. 2026 Mar 5;58(5):1269–82. doi: 10.1002/evj.70156 (PMC13447866; doi:10.1002/evj.70156)
Supplement: Supplementary file 1 [file EVJ-58-1269-s001.pdf]

Table S2: Sensitivity analysis of lag structure for Irish imports (commodity code 010129; non–pure-bred horses) and monthly numbers of equine influenza (EI) outbreaks in the UK, 2020–2024 (restricted to *n* = 58 months with complete lagged import data).

	**Model 2 Irish imports (commodity code 010129) in quartiles with 1-month lag, restricted**		**Model 2a Irish imports (commodity code 010129) in quartiles with 2-month lag**	
**Variable**	**IRR (95% CI)**	**p-value**	**IRR (95% CI)**	**p-value**
**Quarter 1 (January-March)**	1.00	Ref.	1.00	Ref.
**Quarter 2 (April-June)**	1.58 (0.68–3.69)	0.29	2.19 (0.86–5.58)	0.099
**Quarter 3 (July-September)**	1.60 (0.70–3.67)	0.26	2.11 (0.82–5.42)	0.12
**Quarter 4 (October-December)**	3.71 (1.69–8.13)	<0.001***	4.62 (1.90–11.25)	<0.001***
**Group 1 import volume quartile**	1.00	Ref	1.00	Ref.
**Group 2 import volume quartile**	1.19 (0.48–2.97)	0.70	1.54 (0.64–3.69)	0.33
**Group 3 import volume quartile**	4.27 (2.02–9.02)	<0.001***	1.71 (0.75–3.93)	0.20
**Group 4 import volume quartile**	3.53 (1.65–7.53)	0.001**	2.51 (1.10–5.72)	0.028*
**AIC**	213		226	

Notes: $p < 0.05$ (*), $p < 0.01$ (**), $p < 0.001$ (***). P-values are two-sided from negative binomial regression; unstarred values are not significant at $p < 0.05$.
Ref. = reference category; IRR = incidence rate ratio; AIC = Akaike Information Criterion.
