# Supplementary material for: What happened after the epidemic? Equine influenza surveillance sheds light on sources and seasonal risk in the United Kingdom
Source: Equine Vet J. 2026 Mar 5;58(5):1269–82. doi: 10.1002/evj.70156 (PMC13447866; doi:10.1002/evj.70156)
Supplement: Supplementary file 2 [file EVJ-58-1269-s002.pdf]

Equine Veterinary Journal BEVA

Figure S1: Example of an outbreak form used to gather epidemiological data on equine influenza outbreaks in the UK from 2020 to 2024.

Outbreak Information

Attending vet
Vet name.
Practice name.
Practice address.

Telephone.
Email.

Horse location
County. Post Code.

Horse details
Please ensure you fill in **horse details** and **disease information** for **each horse sampled**
Horse Name.
Age/DOB.
Gender ☐ mare/filly ☐ gelding ☐ stallion/colt
Breed/type.

Latest **influenza** vaccination history
Date administered.
Vaccine brand used.
☐ 1st vacc ☐ 6mo booster
☐ 2nd vacc ☐ annual booster
☐ 1st booster ☐ unvaccinated

Latest **EHV** vaccination history
Date administered.
Vaccine brand used.
☐ 1st vacc ☐ 5 months of pregnancy
☐ 2nd vacc ☐ 7 months of pregnancy
☐ 6mo booster ☐ 9 months of pregnancy
☐ unvaccinated

Diagnostic testing
Diagnosis.
Date sample taken.
Date sample confirmed positive.
Sample type(s) submitted.
Test(s) conducted.
Test result (if PCR inc. CT value/copy no.).
Name of receiving lab.

Disease information
TPR.
☐ Inappetence
☐ Lethargy
☐ Ocular discharge
☐ Lymphadenopathy

Cough:
☐ dry/harsh
☐ productive
☐ severe

Neuro signs:
☐ ataxia
☐ hind limb paresis
☐ recumbency
☐ urinary incontience
☐ reduction/loss of anal tone

Nasal discharge:
☐ serous
☐ mucoid
☐ mucopurulent

Abortion (stage of pregnancy).

Other (describe).

What date was the illness first recognised?
How many other horses are affected?
Have others on the premises been tested?
☐ No ☐ Yes Further info.

Often infectious diseases are associated with horse movements, to aid control and prevention measures, please fill in the information below. This section only needs to be filled out once per infected premises

Premises type (please tick most relevant):
☐ Private
☐ Livery
☐ Training/competition yard
☐ Stud
☐ Riding school
☐ Other

Horse kept in (please tick all that apply):
☐ Individual stables
☐ Barn system
☐ Separate grazing
☐ Group grazing
☐ Other

Are isolation facilities available?
☐ No ☐ Yes
Are new arrivals quarantined?
☐ No ☐ Yes
In the last 2 weeks:
Have horses been offsite?
☐ No ☐ Yes
Further info. to shows

Have there been new arrivals?
☐ No ☐ Yes
Further info.

Do horses share:
Tack? ☐ No ☐ Yes
Equipment? ☐ No ☐ Yes

No. of horses on premises. 35
No. of in-contacts with this confirmed case.
Are there pregnant mares on the premises? ☐ No ☐ Yes

Proportion of horses vaccinated for
Influenza:
☐ All ☐ Majority ☐ Half ☐ Minority ☐ None
EHV:
☐ All ☐ Majority ☐ Half ☐ Minority ☐ None

Have infectious disease outbreaks been confirmed on the premises before?
☐ No ☐ Yes
If yes, what and when?
