# Supplementary material for: What happened after the epidemic? Equine influenza surveillance sheds light on sources and seasonal risk in the United Kingdom
Source: Equine Vet J. 2026 Mar 5;58(5):1269–82. doi: 10.1002/evj.70156 (PMC13447866; doi:10.1002/evj.70156)
Supplement: Supplementary file 3 [file EVJ-58-1269-s003.pdf]

Table S1: Models 3–5 examine the effect of import volume from Ireland across three commodity codes—0101 (all horses), 010121 (pure-bred breeding horses), and 010129 (non-purebred horses)—converted into quartile-based groups (Group 1 = lowest, Group 4 = highest volume).

	**Model 3 Irish imports by type: All horses (0101) in quartiles**		**Model 4 Irish imports by type: Pure-bred breeding horses (010121) in quartiles**		**Model 5 Irish imports by type: Non pure-bred (010129) in quartiles**	
**Variable**	**IRR (95% CI)**	**p-value**	**IRR (95% CI)**	**p-value**	**IRR (95% CI)**	**p-value**
**Quarter 1 (January-March)**	1.00	Ref.	1.00	Ref	1.00	Ref
**Quarter 2 (April-June)**	2.69 (0.81-8.91)	0.11	3.86 (1.02-14.67)	0.05	1.95 (0.72-5.32)	0.19
**Quarter 3 (July-September)**	2.53 (0.85-7.59)	0.097	4.09 (1.10-15.22)	0.04*	2.15 (0.85-5.45)	0.11
**Quarter 4 (October-December)**	6.48 (2.16-19.46)	<0.001**	12.58 (3.43-46.09)	<0.001**	4.99 (1.96-12.70)	<0.001***
**Year 2020**	1.00	Ref.	1.00	Ref.	1.00	Ref.
**Year 2021**	5.30 (1.45-19.33)	0.012*	7.87 (2.15-28.81)	<0.001***	3.82 (1.17-12.48)	0.03*
**Year 2022**	4.28 (1.13-16.12)	0.032*	6.53 (1.70-25.13)	0.01**	3.00 (0.85-10.53)	0.09
**Year 2023**	5.00 (1.34-18.66)	0.017*	8.63 (2.17-34.34)	<0.0001***	3.74 (1.11-12.55)	0.03*
**Year 2024**	4.67 (1.23-17.69)	0.023	6.76 (1.86-24.58)	<0.0001***	3.21 (0.92-11.22)	0.07
**Group 1 import volume quartile**	1.00	Ref.	1.00	Ref.	1.00	Ref.
**Group 2 import volume quartile**	1.00 (0.29-3.50)	1.00	0.43 (0.11-1.66)	0.22	1.08 (0.39-3.01)	0.89
**Group 3 import volume quartiles**	1.02 (0.26-4.00)	0.98	0.56 (0.14-2.29)	0.42	1.93 (0.68-5.48)	0.22

**Group 4 import volume quartiles**	1.15 (0.30-4.39)	0.84	0.39 (0.09-1.64)	0.20	1.44 (0.49-4.22)	0.50
**AIC**	228		226		226	

Notes: $p < 0.05$ (*), $p < 0.01$ (**), $p < 0.001$ (***). P-values are two-sided from negative binomial regression; unstarred values are not significant at $p < 0.05$. Ref. = reference category; IRR = incidence rate ratio; AIC = Akaike Information Criterion.
