# Supplementary material for: What happened after the epidemic? Equine influenza surveillance sheds light on sources and seasonal risk in the United Kingdom
Source: Equine Vet J. 2026 Mar 5;58(5):1269–82. doi: 10.1002/evj.70156 (PMC13447866; doi:10.1002/evj.70156)
Supplement: Supplementary file 4 [file EVJ-58-1269-s004.pdf]

Equine Veterinary Journal BEVA

Table S3: Frequency data for breed type of UK equine influenza cases occurring between 2020 to 2024.

General breed	Breed	Total	Percentage (%)
Undefined	Undefined	28	18.8
UK native horse breeds	Cob	24	16.1
Sports horses	Irish Sports Horse	20	13.4
UK native pony breeds	Shetland Pony	14	9.4
Sports horses	Warmblood	11	7.4
UK native pony breeds	Welsh Pony	11	7.4
UK native pony breeds	Connemara	9	6.0
UK native horse breeds	Irish	4	2.7
Non UK native horses	Andalusian	3	2.0
Sports horses	Thoroughbred	3	2.0
UK native pony breeds	Miniature Shetland Pony	3	2.0
Non UK native horses	Friesian	2	1.3
UK native horse breeds	Clydesdale	2	1.3
UK native horse breeds	Irish Draught	2	1.3
Non UK native horses	Spanish	1	0.4
Sports horses	Pony	1	0.4
Sports horses	Sports Horse	1	0.4
UK native horse breeds	Dartmoor	1	0.4
UK native horse breeds	Shire Horse	1	0.4
UK native pony breeds	Highland	1	0.4
UK native pony breeds	Fell Pony	1	0.4
Crossbreeds	Cross	1	0.4
Crossbreeds	Cross Cob	1	0.4
Crossbreeds	Cross Thoroughbred	1	0.4
Crossbreeds	Cross Trotter	1	0.4
Crossbreeds	Cross Welsh Cob	1	0.4
Donkey	Donkey	1	0.4
