# Supplementary material for: What happened after the epidemic? Equine influenza surveillance sheds light on sources and seasonal risk in the United Kingdom
Source: Equine Vet J. 2026 Mar 5;58(5):1269–82. doi: 10.1002/evj.70156 (PMC13447866; doi:10.1002/evj.70156)

Equine Veterinary Journal BEVA

**Figure S2:** Flow diagram showing how 177 laboratory-confirmed EI-positive samples (2020–2024) relate to 149 confirmed cases with epidemiological data, 126 EI-infected premises, and the subsets used for premises-level and movement-linked analyses.

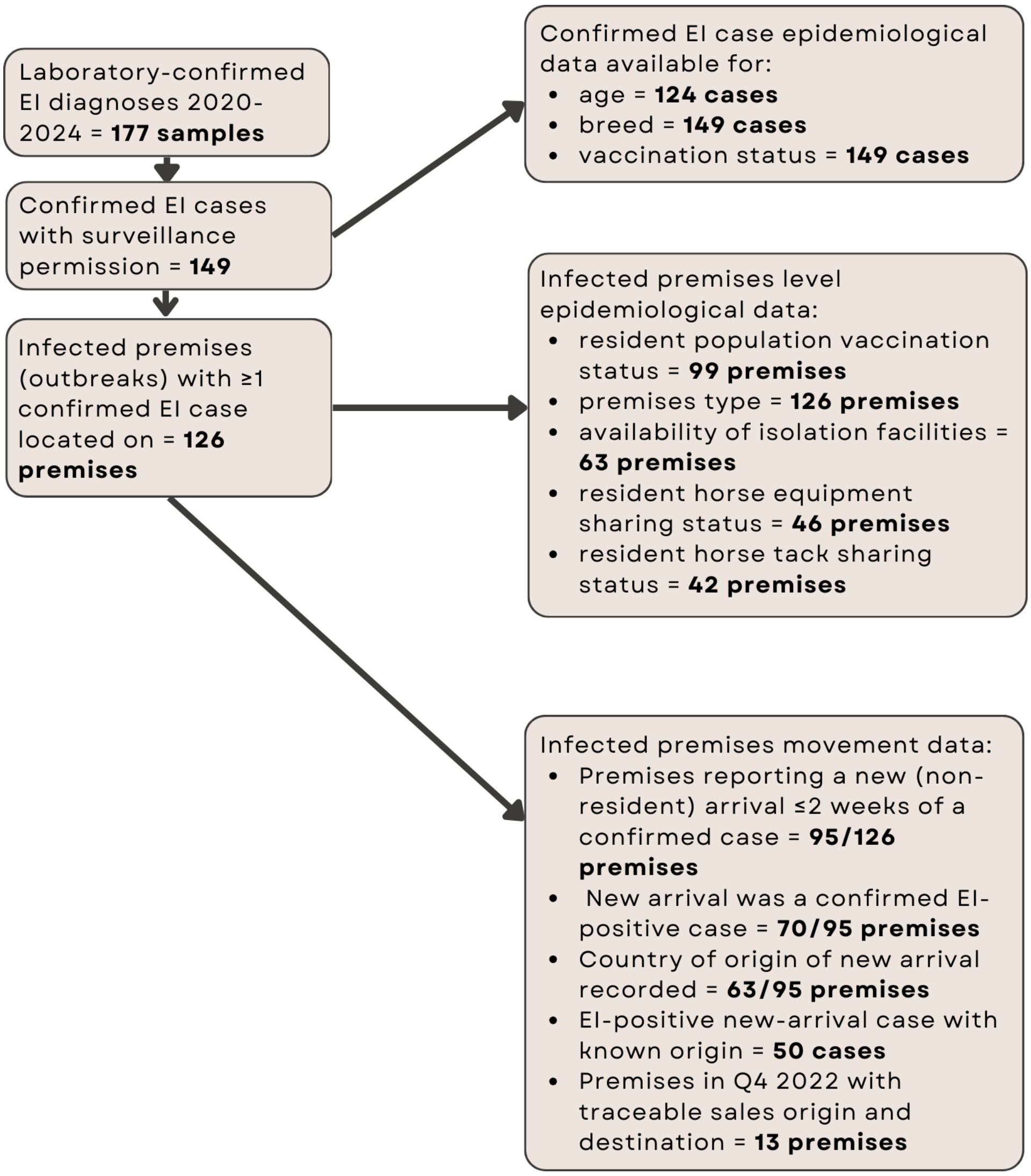

Supplement: Supplementary file 5 [file EVJ-58-1269-s005.pdf]
